# Thalamic, hippocampal and basal ganglia pathology in primary lateral sclerosis and amyotrophic lateral sclerosis: Evidence from quantitative imaging data

**DOI:** 10.1016/j.dib.2020.105115

**Published:** 2020-01-10

**Authors:** Eoin Finegan, Stacey Li Hi Shing, Rangariroyashe H. Chipika, Mary C. McKenna, Mark A. Doherty, Jennifer C. Hengeveld, Alice Vajda, Colette Donaghy, Russell L. McLaughlin, Siobhan Hutchinson, Orla Hardiman, Peter Bede

**Affiliations:** a Computational Neuroimaging Group, Biomedical Sciences Institute, Trinity College Dublin, 152-160 Pearse Street, Dublin 2, Ireland; b Complex Trait Genomics Laboratory, Smurfit Institute of Genetics, Trinity College Dublin, 1-5 College Green, Dublin 2, Ireland; c Western Health & Social Care Trust, Belfast, UK; d Department of Neurology, St James's Hospital, James's St, Ushers, Dublin 8, D08 NHY1, Ireland

**Keywords:** Primary lateral sclerosis, Amyotrophic lateral sclerosis, Neuroimaging, MRI, Thalamus, Hippocampus, Basal ganglia, Biomarkers

## Abstract

Primary lateral sclerosis and amyotrophic lateral sclerosis are primarily associated with motor cortex and corticospinal tract pathology. A standardised, prospective, single-centre neuroimaging protocol was used to characterise thalamic, hippocampal and basal ganglia involvement in 33 patients with primary lateral sclerosis (PLS), 100 patients with amyotrophic lateral sclerosis (ALS), and 117 healthy controls. “Widespread subcortical grey matter degeneration in primary lateral sclerosis: a multimodal imaging study with genetic profiling” [1] Imaging data were acquired on a 3 T MRI system using a 3D Inversion Recovery prepared Spoiled Gradient Recalled echo sequence. Model based segmentation was used to estimate the volumes of the thalamus, hippocampus, amygdala, caudate, pallidum, putamen and accumbens nucleus in each hemisphere. The hippocampus was further parcellated into cytologically-defined subfields. Total intracranial volume (TIV) was estimated for each participant to aid the interpretation of subcortical volume alterations. Group comparisons were corrected for age, gender, TIV, education and symptom duration. Considerable thalamic, hippocampal and accumbens nucleus atrophy was detected in PLS compared to healthy controls and selective dentate, molecular layer, CA1, CA3, and CA4 hippocampal pathology was also identified. In ALS, additional volume reductions were noted in the amygdala, left caudate and the hippocampal-amygdala transition area of the hippocampus. Our imaging data provide evidence of extensive and phenotype-specific patterns of subcortical degeneration in PLS.

**Table:** Specifications Table

Subject	Primary Lateral Sclerosis, Radiology, Neuroimaging
Specific subject area	MRI, Grey matter volumetry, Hippocampus
Type of data	Volumetric neuroimaging data with standardised acquisition
How data were acquired	Imaging data were acquired on a Philips Achieva 3T MRI scanner (Philips Medical Systems, Best, The Netherlands) with an 8-channel head coil.
Data format	Estimated marginal means and standard error for subcortical grey matter structures and hippocampal subfields adjusted for total-intracranial volume, age, gender, and education.
Parameters for data collection	3D–T1-weighted sequence: spatial resolution: 1 × 1 × 1 mm, Field of view: 256 × 256 × 160 mm, TR/TE = 8.5/3.9 ms, TI = 1060 ms, flip angle = 8°, SENSE factor = 1.5, sagittal acquisition; 256 slices.
Description of data collection	The protocol, consent forms, recruitment procedures, and data management were approved by the institutional ethics committee. All participants provided informed consent prior to inclusion. Participating ALS patients were diagnosed according to the El Escorial research criteria and PLS patients were diagnosed according to the Gordon criteria. Patients underwent standardised neurological assessments and MRI data were acquired with uniform pulse sequence settings and anonymised.
Data source location	Institution: Computational neuroimaging group, Trinity Biomedical Sciences Institute, Trinity College Dublin City/Town/Region: Dublin Country: Ireland
Data accessibility	The subcortical grey matter profile and hippocampal subfield features of the three groups are presented as raw data in box plots and contrasted in statistical tables using the relevant covariates.
Related research article	Authors: Eoin Finegan, Stacey Li Hi Shing, Rangariroyashe H. Chipika, Mark A. Doherty, Jennifer C. Hengeveld, Alice Vajda, Colette Donaghy, Niall Pender, Russell L. McLaughlin, Orla Hardiman, Peter Bede Title: Widespread subcortical grey matter degeneration in primary lateral sclerosis: a multimodal imaging study with genetic profiling Journal: Neuroimage Clinical DOI: https://doi.org/10.1016/j.nicl.2019.102089

**Table:** 

**Value of the Data** - This dataset confirms extensive extra-motor involvement in primary lateral sclerosis (PLS) - The data reveal evidence of considerable thalamic, hippocampal, accumbens, amygdala and caudate atrophy in PLS - The data confirm divergent subcortical imaging signatures in ALS and PLS - The presented data may guide future post mortem studies to characterise pTDP-43 load in subcortical grey matter structures.

## 1 Data

**Fig. 1:**
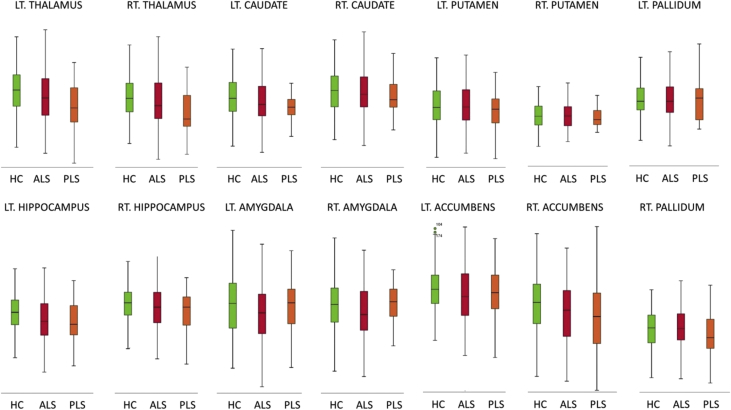
Subcortical grey matter volumes in primary lateral sclerosis (PLS), amyotrophic lateral sclerosis (ALS) and healthy controls (HC).

**Fig. 2:**
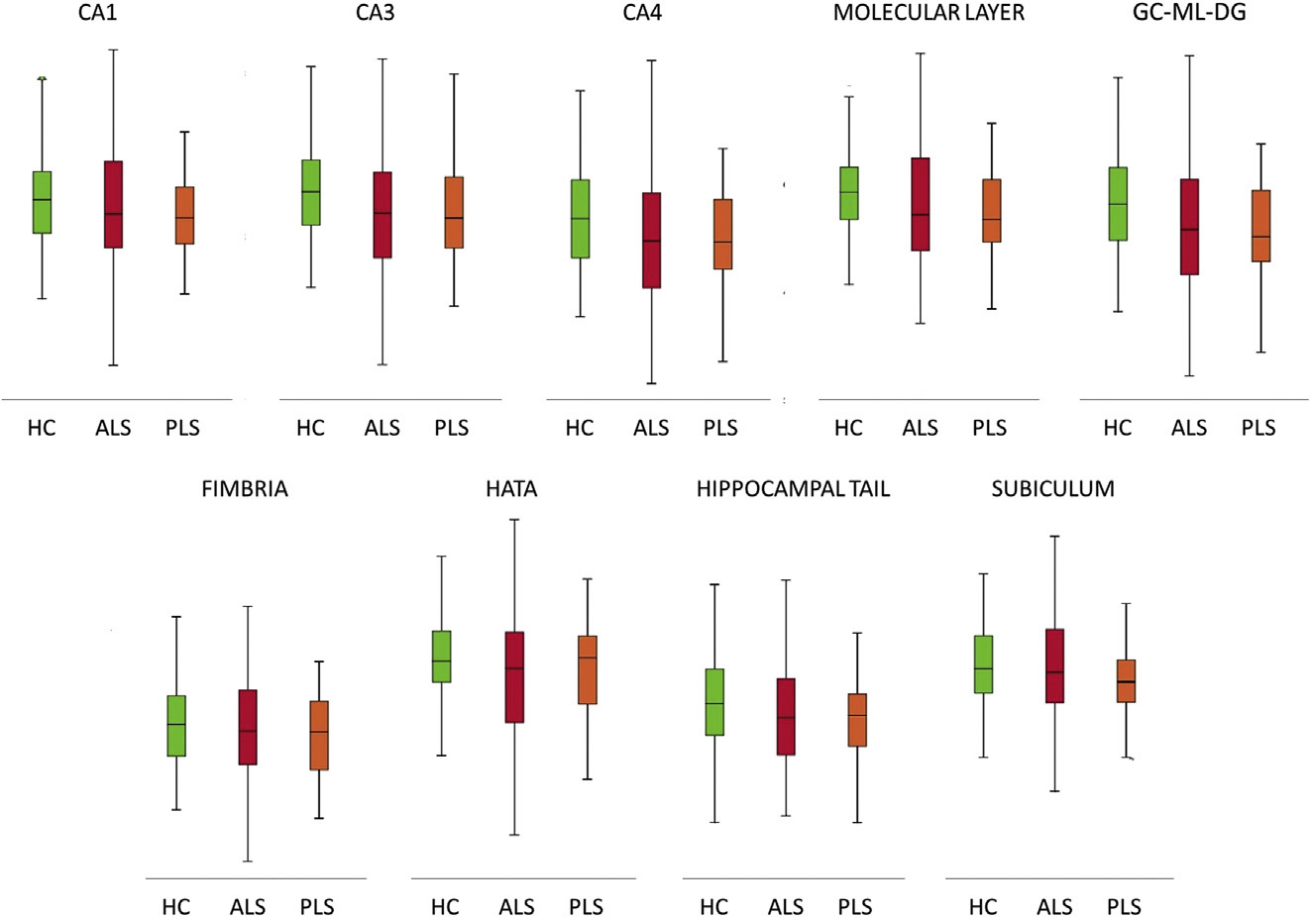
Hippocampal subfield volumes in primary lateral sclerosis (PLS), amyotrophic lateral sclerosis (ALS) and healthy controls (HC). CA = Cornu Ammonis; GC-ML-DG = Granule Cell and Molecular Layer of the Dentate Gyrus; HATA = hippocampus-amygdala transition area.

**Fig. 3:**
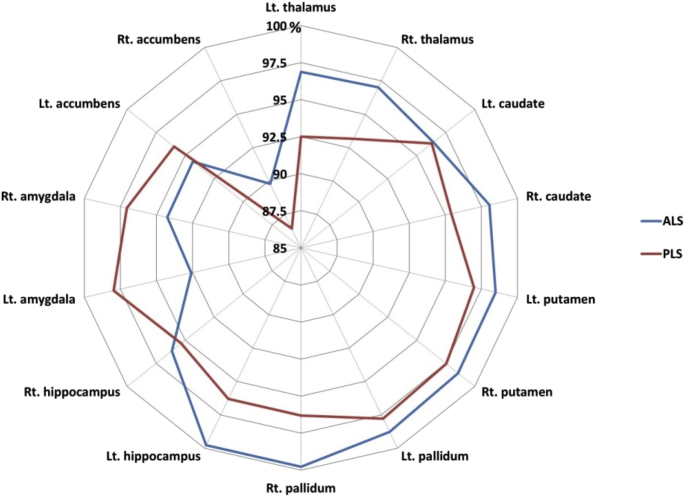
The subcortical volumetric profile of PLS and ALS with reference to healthy controls. Estimated marginal means of volumes were calculated for each structure with the following values age = 58.77, gender = 1.45, education = 13.78, and total intracranial volume = 1429699.38. The estimated marginal means of healthy controls represent 100%.

**Fig. 4:**
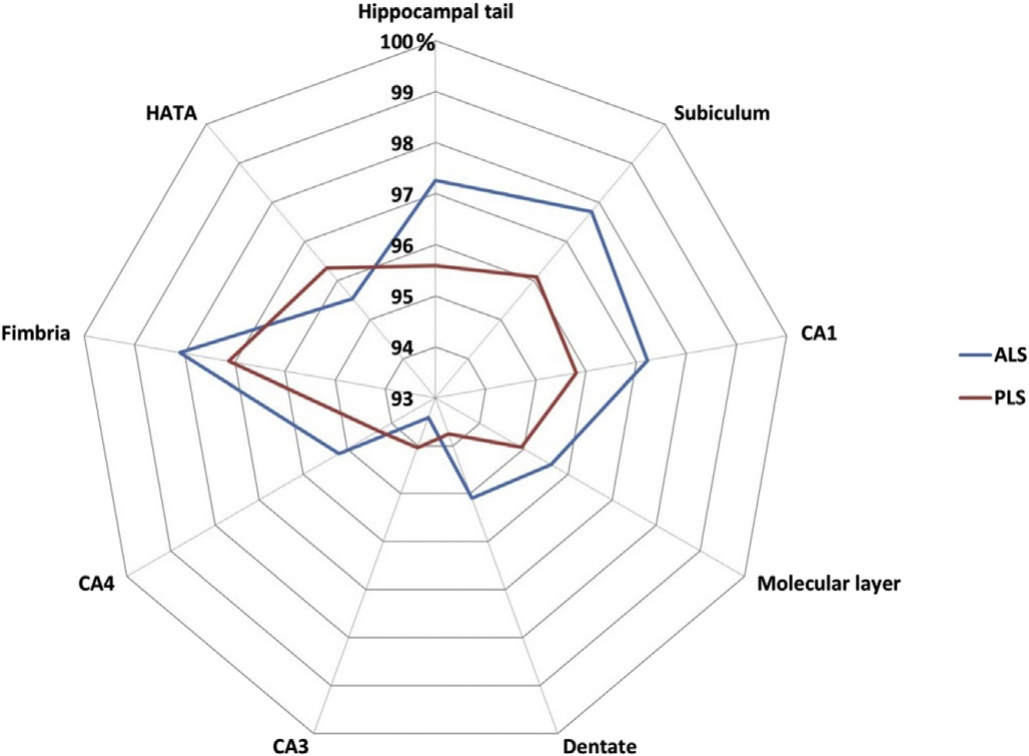
The hippocampal volumetric profile of PLS and ALS with reference to healthy controls. Estimated marginal means of volumes were calculated for each structure with the following values age = 58.77, gender = 1.45, education = 13.78, and total intracranial volume = 1429699.38. The estimated marginal means of healthy controls represent 100%.

The majority of primary lateral sclerosis and amyotrophic lateral sclerosis studies focus on the motor cortex and corticospinal tracts [[2], [3], [4], [5]]. In this dataset (Table 1) we present total intracranial volume, thalamus, hippocampus, amygdala, caudate, pallidum, putamen and accumbens nucleus volumes for 33 patients with primary lateral sclerosis, 100 patients with amyotrophic lateral sclerosis and 117 age and gender-matched healthy controls. Data for bilateral structures are presented separately. Additionally, we provide hippocampal subfield volume estimates for the CA1, CA2/3, CA4, fimbria, subiculum, hippocampal tail, molecular layer, dentate gyrus, and hippocampal-amygdala transition area (HATA). Accompanying clinical and demographic characteristics are presented to highlight that the patient groups were matched for age, gender and ALSFRS-r and differed in years of education and symptom duration. Raw subcortical volumetric data (Fig. 1) and raw hippocampal subfield characteristics (Fig. 2) are presented in box plots for each cohort. The estimated marginal means and standard error of subcortical structures adjusted for the relevant clinical, radiological and demographic variables (age, gender, education, total intracranial volume, symptom duration) are summarised in Table 2, and hippocampal subfield profiles are presented in Table 3 using the same covariates. Based on estimated marginal means corrected for age, gender, total intracranial volumes and education, the comparative profile of the two patient groups are also illustrated in radar plots with reference to healthy controls. Contrary to our original paper [1] where percentage change was presented as absolute values, here, ‘100%’ represents the estimated marginal means of healthy controls as normative values and the concentric circles depict the selective atrophy profiles of the ALS and PLS cohorts (Fig. 3, Fig. 4).

**Table 1:** Data categories and measures ALS = amyotrophic lateral sclerosis; ALSFRS-R = amyotrophic lateral sclerosis functional rating scale-revised; PLS = Primary lateral sclerosis; CA = Cornu Ammonis; GC-DG = granule cell layer of the dentate gyrus; HATA = hippocampus-amygdala transition area, Lt = Left, Rt = Right.

Data categories	Specific measures
Demographic variables	Age (year)
	Gender (Male/Female)
	Years of education (years)
	Handedness (Rt/Lt)
Clinical data for ALS and PLS	Symptom duration (months)
	ALSFRS-R (max 48)
Subcortical grey matter structure volumes	hippocampus (mm^3^)
	amygdala (mm^3^)
	thalamus (mm^3^)
	nucleus accumbens (mm^3^)
	caudate nucleus (mm^3^)
	putamen (mm^3^)
	pallidum (mm^3^)
Hippocampal subfield volumes	CA1 (mm^3^)
	CA2/CA3 (mm^3^)
	CA4 (mm^3^)
	Fimbria (mm^3^)
	Subiculum (mm^3^)
	Molecular layer (mm^3^)
	GC-ML-DG (mm^3^)
	HATA (mm^3^)

**Table 2:** The demographic, clinical and subcortical grey matter profile of the three groups. ALSFRS-r = the revised ALS functional rating scale, EMM = estimated marginal mean, M = Mean, N/a = Not applicable, SE = standard error, SD = standard deviation. Estimate marginal means are adjusted with the following values age = 58.77, gender = 1.45, education = 13.78, and total intracranial volume = 1429699.38.

	PLS n = 33	ALS n = 100	Healthy Controls n = 117	p value
Age M/SD	60.5 (10.5)	59.8 (11.2)	57.4 (11.9)	0.19
Gender (M/F)	19/14	62/38	56/61	0.11
Education (years) M/SD	12.9 (3.4)	13.5 (3.2)	14.3 (3.3)	0.04
Handedness (R/L)	29/4	90/10	109/8	0.55
ALSFRS-r (max 48) M/SD	34.4 (5.3)	36.6 (7.5)	N/a	0.11
Symptom Duration (months) M/SD	121.76 (68.7)	20.6 (14.3)	N/a	7.1E-16
Total intracranial volume (mm^3^) M/SD	1439675.5 (145614.1)	1440623.0 (149085.7)	1417549.2 (128172.3)	0.432
Hippocampus (Left) EMM/SE	3566.6 (84.4)	3624.1 (48.5)	3805.1 (45.1)	0.007
Hippocampus (Right) EMM/SE	3707.4 (85.4)	3739.1 (49.1)	3888.5 (45.7)	0.045
Amygdala (Left) EMM/SE	1189.4 (42.3)	1124.0 (24.4)	1213.9 (22.7)	0.03
Amygdala (Right) EMM/SE	1144.2 (44.0)	1111.2 (25.3)	1178.8 (23.5)	0.16
Thalamus (Left) EMM/SE	7044.0 (97.9)	7376.1 (56.3)	7614.0 (52.4)	2E-6
Thalamus (Right) EMM/SE	6896.5 (81.5)	7181.9 (52.6)	7402.8 (48.9)	5E-6
Nucleus accumbens (Left) EMM/SE	473.8 (19.6)	465.8 (11.3)	493.8 (10.5)	0.19
Nucleus accumbens (Right) EMM/SE	326.9 (19.0)	339.6 (10.9)	378.2 (10.2)	0.01
Caudate nucleus (Left) EMM/SE	3283.3 (60.0)	3287.5 (34.5)	3409.6 (32.1)	0.02
Caudate nucleus (Right) EMM/SE	3412.4 (66.3)	3506.8 (38.1)	3576.7 (35.5)	0.08
Putamen (Left) EMM/SE	4550.8 (82.3)	4620.7 (47.3)	4692.2 (44.0)	0.27
Putamen (Right) EMM/SE	4625.6 (86.7)	4674.1 (50.0)	4741.9 (46.3)	0.41
Pallidum (Left) EMM/SE	1733.7 (36.3)	1751.6 (20.8)	1773.1 (19.4)	0.58
Pallidum (Right) EMM/SE	1713.7 (40.5)	1775.4 (23.3)	1779.5 (21.7)	0.34

**Table 3:** The hippocampal profile of the three groups. ALSFRS-r = the revised ALS functional rating scale, EMM = estimated marginal mean, M = Mean, N/a = Not applicable, SE = standard error, SD = standard deviation. Estimate marginal means are adjusted with the following values age = 58.77, gender = 1.45, education = 13.78, and total intracranial volume = 1429699.38.

	PLS n = 33	ALS n = 100	Healthy Controls n = 117	p value
CA1 (mm^3^) EMM/SE	632.8 (10.8)	642.2 (6.2)	660.5 (5.8)	0.03
CA2/CA3 (mm^3^) EMM/SE	214.1 (5.0)	212.6 (2.9)	227.6 (2.7)	5.2E-4
CA4 (mm^3^) EMM/SE	254.6 (4.6)	257.0 (2.6)	270.0 (2.5)	4.2E-4
Fimbria (mm^3^) EMM/SE	74.9 (2.9)	75.6 (1.6)	77.1 (1.5)	0.72
Subiculum (mm^3^) EMM/SE	421.6 (7.4)	428.9 (4.3)	438.7 (4.0)	0.08
Molecular layer (mm^3^) EMM/SE	559.5 (9.3)	563.5 (5.4)	589.3 (5.0)	6.5E-4
GC-ML-DG (mm^3^) EMM/SE	292.3 (5.4)	296.5 (3.1)	311.8 (2.9)	2.9E-4
HATA (mm^3^) EMM/SE	62.7 (1.5)	62.2 (0.9)	65.1 (0.8)	0.04

## 2 Experimental design, materials, and methods

Following institutional ethics approval, patients were recruited from a national motor neuron disease clinic and data were acquired with a standardised protocol [6]. Participating ALS patients were diagnosed according to the El Escorial research criteria and PLS patients were diagnosed according to the Gordon criteria. The protocol was specifically designed to characterise subcortical grey matter degeneration in PLS based on evidence of extra-motor involvement in other motor neuron diseases [[7], [8], [9], [10], [11]]. T1-weighted images were acquired with a spatial resolution of 1 × 1 × 1mm and field of view of 256 × 256 × 160 mm using a 3D Inversion Recovery prepared Spoiled Gradient Recalled echo (IR-SPGR) sequence. Pulse sequence settings are as follows: repetition time (TR) = 8.5 ms, echo time (TE) = 3.9 ms, Inversion time (TI) = 1060 ms, flip angle = 8°, SENSE factor = 1.5 [12]. Raw MRI data underwent thorough quality control before pre-processing. Following ‘deskulling’ and spatial registration, model based segmentation was used the estimate subcortical volumes using FSL-FIRST of the FMRIB's Software Library (FSL) [13,14]. Raw volumetric data were recorded for the hippocampus, amygdala, thalamus, nucleus accumbens, caudate nucleus, putamen, and pallidum in each hemisphere. Subsequently, the hippocampus of each participant was segmented into cytologically-defined subfields using version 6.0 of the FreeSurfer image analysis suite to estimate volumes of the CA1, CA2/3, CA4, fimbria, subiculum, hippocampal tail, molecular layer, dentate gyrus, and the hippocampal-amygdala transition area [15]. Analyses of covariance (ANCOVA) were used to explore intergroup volumetric differences using age, education, gender and TIV as covariates [16,17]. PLS versus ALS contrasts were also corrected for symptom duration. To illustrate disease-specific volumetric traits in PLS and ALS, the estimated marginal means of each structure were plotted on radar charts with reference to healthy controls.
